# CHARACTERISTICS OF OLDER ADULTS' LEISURE ACTIVITIES: A DAILY DIARY INVESTIGATION

**DOI:** 10.1093/geroni/igac059.803

**Published:** 2022-12-20

**Authors:** Junyan Tian, Abigail Stephan, Lesley Ross

**Affiliations:** Penn State University, State College, Pennsylvania, United States; Clemson University, Clemson, South Carolina, United States; Clemson University, Clemson, South Carolina, United States

## Abstract

Leisure activity engagement in older adults’ daily lives is well-documented, however, relatively few studies include participant-reported activities using prospective daily diary methods. The goal of the study was to examine the proportion of three types of reported leisure activities and how physical health moderated such activities. Two weeks of self-reported leisure activities examined among healthy sedentary community-dwelling adults from the Cognitive and Physical Exercise Study (N=33, 65-81 years, 442 unique data points). Each day, participants listed leisure activities performed, estimated time spent on each activity, and labeled the activities as cognitive, physical, and/or social. Physical health was assessed by the SF-12 at baseline. Across 14 days, 25.14% of reported activities were labeled as cognitive activities, 26.61% were labeled as social activities, and 22.97% were labeled as physical activities. Multilevel models indicated participants’ engagement in different domains of activities were not influenced by physical health and did not change over time.

